# Mono-Heteromeric Configurations of Gap Junction Channels Formed by Connexin43 and Connexin45 Reduce Unitary Conductance and Determine both Voltage Gating and Metabolic Flux Asymmetry

**DOI:** 10.3389/fphys.2017.00346

**Published:** 2017-05-29

**Authors:** Guoqiang Zhong, Nazem Akoum, Daniel A. Appadurai, Volodya Hayrapetyan, Osman Ahmed, Agustin D. Martinez, Eric C. Beyer, Alonso P. Moreno

**Affiliations:** ^1^Department of Cardiology, First Affiliated Hospital of Guangxi Medical UniversityGuangxi, China; ^2^University Medical Center, University of WashingtonSeattle, WA, United States; ^3^Huntsman Institute, University of UtahSalt Lake City, UT, United States; ^4^Health Science Center, University of TexasTexas, TX, United States; ^5^Atlanta Heart SpecialistsAtlanta, GA, United States; ^6^Centro Interdisciplinario de Neurociencia de Valparaíso, Facultad de Ciencias, Universidad de ValparaísoValparaíso, Chile; ^7^Department of Pediatrics, University of ChicagoChicago, IL, United States; ^8^Cardiovascular Research and Training Institute (CVRTI), Department of Bioengineering, University of UtahSalt Lake Citiy, UT, United States

**Keywords:** intercellular communication, heteromeric connexons, gap junctions, permeability, protein kinase C

## Abstract

In cardiac tissues, the expression of multiple connexins (Cx40, Cx43, Cx45, and Cx30.2) is a requirement for proper development and function. Gap junctions formed by these connexins have distinct permeability and gating mechanisms. Since a single cell can express more than one connexin isoform, the formation of hetero-multimeric gap junction channels provides a tissue with an enormous repertoire of combinations to modulate intercellular communication. To study further the perm-selectivity and gating properties of channels containing Cx43 and Cx45, we studied two monoheteromeric combinations in which a HeLa cell co-transfected with Cx43 and Cx45 was paired with a cell expressing only one of these connexins. Macroscopic measurements of total conductance between cell pairs indicated a drastic reduction in total conductance for mono-heteromeric channels. In terms of Vj dependent gating, Cx43 homomeric connexons facing heteromeric connexons only responded weakly to voltage negativity. Cx45 homomeric connexons exhibited no change in Vj gating when facing heteromeric connexons. The distributions of unitary conductances (γj) for both mono-heteromeric channels were smaller than predicted, and both showed low permeability to the fluorescent dyes Lucifer yellow and Rhodamine123. For both mono-heteromeric channels, we observed flux asymmetry regardless of dye charge: flux was higher in the direction of the heteromeric connexon for MhetCx45 and in the direction of the homomeric Cx43 connexon for MhetCx43. Thus, our data suggest that co-expression of Cx45 and Cx43 induces the formation of heteromeric connexons with greatly reduced permeability and unitary conductance. Furthermore, it increases the asymmetry for voltage gating for opposing connexons, and it favors asymmetric flux of molecules across the junction that depends primarily on the size (not the charge) of the crossing molecules.

## Introduction

Connexins constitute a homologous family of proteins that oligomerize to form gap junction channels that allow communication between cells. Six connexins oligomerize to form gap junction connexons (hemi-channels). A connexon docks with another connexon within the plasma membrane of an adjacent cell to from a complete gap junction channel.

Like many other organs, the heart, expresses multiple connexins with different functions including connexin43 (Cx43), connexin40 (Cx40), connexin45 (Cx45), and Cx 30.2 (Gros and Jongsma, 1996). These four proteins have been detected in the working and conductive regions of the heart (Bukauskas et al., 2006); however, Cx45 is more abundant in the sinoatrial node (site of impulse generation) and the atrioventricular node (Coppen et al., 1999b,a). Cx40, Cx43, and Cx45 are phospho-proteins, and phosphorylation has been implicated in channel assembly (Paulson et al., 2001), gating of gap junctional communication (Moreno et al., 1992) and regulation of connexin degradation. Abnormal abundances and distributions of these connexins may contribute to cardiac pathologies such as arrhythmias (Saffitz et al., 1999; Thibodeau et al., 2010; Askar et al., 2012).

Each of the cardiac connexins forms channels with unique electrophysiological and regulatory properties as demonstrated when the channels formed by these connexins were studied individually in a homomeric-homotypic configuration, using exogenous expression systems like transfected tumor cells or *Xenopus* oocytes. Cardiac connexin gap junction channels have different transjunctional voltage dependence, charge selectivity, and unitary conductance (Moreno et al., 1995a; Musa et al., 2001; Rackauskas et al., 2007).

More than one connexin can be expressed by the same cell and within the same junction (Beyer et al., 2001), and different connexins can associate to form hetero-oligomeric junctional channels. Formation of channels from hetero-oligomeric combinations of connexins with different intrinsic properties may provide novel ways to control cell-to-cell communication between different cells in a tissue.

Our laboratories and others have presented strong evidence that heteromeric connexons can be formed in cardiac and non-cardiac tissues (He and Burt, 1998; Valiunas et al., 2001; Cottrell et al., 2002; Martinez et al., 2002; Koval et al., 2014; Tong et al., 2015). Nonetheless, to understand fully the consequences of these oligomeric interactions, it has been necessary to use cellular systems in which the expression and assembly of hemichannels among different cells can be induced.

The simplest hetero-multimeric configuration is the “heterotypic” combination of homomeric connexons, where all subunits of connexons on one side of the channel are identical and the opposing connexons are formed by a different connexin. The natural presence and functional consequences of such heterotypic channels were proposed many years ago (Loewenstein, 1981; Ramon and Rivera, 1986), and some of the properties of heterotypic combinations have been studied more recently in *Xenopus* oocytes (Rubin et al., 1992; Nicholson et al., 1993; White et al., 1995), transfected SkHep1 (Moreno et al., 1995b), HeLa (Elfgang et al., 1995), or N2A cells (Elenes et al., 2001; Bukauskas et al., 2002).

A more complicated situation arises when one connexon is formed of more than one connexin isoform (“heteromeric” connexon). These new arrangements open the possibility of regulating the properties of the intercellular junctions depending on the amount, spatial distribution and interrelations of all connexins co-expressed. In addition, formation of heteromeric channels has been implicated in pathological conditions, like syndromic deafness caused by connexin mutants (García et al., 2015). In a previous report, we have shown biochemical and electrophysiological evidence that Cx45 and Cx43 can form heteromeric connexons in a bi-heteromeric configuration, with novel permeability, and gating properties (Martinez et al., 2002).

Cardiocytes in the sinoatrial and atrioventricular node cells express mainly Cx45, but in the surrounding working heart the cardio-myoblast cells express a combination of Cx43 and Cx45; hence, it is highly probable that mono-heteromeric channels can be generated in the transition zone (edges) between these two tissues. However, this type of channels has not been characterized yet. Therefore, to dissect further the effects of oligomerization between Cx43 and Cx45 upon unitary and total conductance, gating produced by transjunctional voltage, and permeability properties, we studied “mono-heteromeric” combinations of channels where one side of the junction contained only homomeric connexons while the other had multiple heteromeric possible combinations. Some previous studies of pairs of cells co-expressing two connexins have suggested that properties of Bi-heteromeric channels (like unitary conductance or gating) are intermediate between those of the two corresponding homomeric channels (Brink et al., 1997; Beyer et al., 1998; He and Burt, 1998; Martinez et al., 2002). Our results with mono-heteromeric channels indicate that if heteromerization occurs randomly in each connexon, its unitary conductance results smaller than the predicted values.

## Methods

### Cell culture

HeLa cells transfected with rat Cx43 containing a carboxyl terminal (His)6 epitope, with chicken Cx45, or with both connexins have been described and characterized previously [Cx43: Accession X06656 M19317; Cx45 Accession NM 205503(Martinez et al., 2002)]. HeLaCx43 or HeLaCx45 cells were co-cultured at a ratio of 1:1 with the co-transfected HeLaCx43/Cx45 cells allowing the formation of mono-heteromeric channels with Cx43 (MhetCx43) and Cx45 (MhetCx45) homomeric connexons, respectively. In this manuscript, we will refer to rat Cx43(His)6 and chicken Cx45 as Cx43 and Cx45. Rat (Rattus norvergicus) Cx43 and chicken (Gallus gallus) Cx45 present 83% identity. The main differences are located at the intracellular loop and at the CT domain. The first extracellular loops have 100% identity. The second extracellular loops have 85% identity, with no differences in critical residues relevant for stabilization of docking connexons. For discrimination under epifluorescence microscopy, co-transfected cells expressing the heteromeric connexons were stained with 100 μM DiI Red (Molecular Probes Inc. D-282), while cells that expressed Cx45 or Cx43 were loaded with 50 μM Cell Tracker Green (Molecular Probes Inc. C-2925), both for 50 min at 37°C. To identify injected cells after fixation, cells were plated on coverslips with a central etched grid (Cell-Locator, CA).

### Immunoblotting

Samples of parental or stably transfected HeLa cells were prepared for immunoblotting. Cell cultures were rinse with PBS (pH 7.4) and then harvest in ice-cold 2 Mm phenylmethylsulfonyl fluoride (PMSF) in PBS. Cell suspensions were centrifuged, the supernatant was discarded, and the pellets were frozen in liquid nitrogen and stored at −80°C. Cell pellets were re-suspended in water containing protease inhibitors (200 mg/mL soybean trypsin inhibitor, 1 mg/mL benzamidine, 1 mg/mL aminocaproic acid, and 2 mM PMSF) and phosphatase inhibitors (20 mM Na4P2O7 and 100 mM NaF) and lysed by sonication. Western blots analysis was perform essentially as described previously (Martinez et al., 2002). Protein samples (100 μg or 50–100 μL of biotynilated, non-biotynilated, and Triton X-100 soluble or insoluble cellular fractions) were resolved on 8 or 10% polyacrylamide gels containing sodium dodecyl sulfate (SDS-PAGE). Proteins were electro-transferred from gels onto Immobilon-P membranes (Millipore, Bedford, MA) at 300 mA for 1.5 h. Membranes were incubated in 5% nonfat milk in Tris-buffered saline (TBS) 0.1% Tween-20, pH 7.4, overnight at 4°C, and then incubated with mouse monoclonal anti-Cx43 (Sigma; C6219) or rabbit polyclonal anti-Cx43 (Chemicon/Millipore; AB1727), and mouse monoclonal anti-Cx45 antibodies (Chemicon/Millipore; MAB3100) diluted in 5% nonfat milk in TBS 0.1% Tween-20 for 3 h at room temperature. Membranes were rinsed repeatedly in TBS 0.1% Tween-20 and then incubated for 30 min at room temperature with horseradish-peroxidase-conjugated secondary goat anti-mouse antibodies (Jackson ImmunoResearch, West Grove, PA). After rinse repeatedly in TBS 0.1% Tween-20, the antibody binding was detected by chemiluminescence (ECL, western blotting detection reagent according to the manufacturer's instructions, Amersham; GE Healthcare Life Science) followed by exposure to imagine system (UVP Vision Works).

### Dye injections

To determine if there was a correlation between the low conductance of mono-heteromeric channels and their permeability to macromolecules, we injected cells within groups that formed mono-heteromeric channels with their neighbors. To identify cells expressing different connexins, they were stained with either Cell Tracker Green (Molecular Probes Inc. C-2925; 50 μM for 50 min) or DiI (Aldrich 468495; 100 μm for 50 min) and then co-cultured at a ratio that allowed one cell of a kind be surrounded by cells from the other kind. Cells were injected using a 20–30 MΩ glass micropipette loaded with the distinct permeability probes (see below). The permeability for each probe was assessed by determining the coupling coefficient (CR) calculated as the ratio of number of first-order dye coupled cells over the number of cells in visible contact with the injected cell.

### Electrophysiology

The dual whole-cell voltage clamp technique was applied to cell pairs to measure their junctional conductance (g_j_). Patch solution: (in mmol/L: 130 CsCl_2_; 0.5 CaCl_2_; 2 Na_2_ATP; 3 MgATP; 10 HEPES; 10 EGTA; pH 7.2). Recording solution: (in mmol/L: 130 NaCl, 7 CsCl_2_, 2.0 CaCl_2_, 0.6 MgCl_2_, 10 HEPES, pH 7.4). The junctional voltage sensitivity was determined by measuring transjunctional current (I_j_) in one of the cells (held at constant zero voltage), while voltage steps (ranging from −100 to 100 mV, incrementing by 20 mV) were applied to its contiguous partner through pClamp7 software (Axon Instruments, CA). Parameters of voltage dependence were determined by normalizing the steady state current (I_ss_) levels during long voltage pulses with regard to the instantaneous current (I_inst_) measured at the beginning of the pulses; normalized conductances are designated by G. The normalized steady-state voltage sensitivity profile (G_ss_/G_i_ − V_j_) was fitted using the equation ${\text{G}}_{\text{s}}{\text{S}/\text{G}}_{\text{i}}={\text{G}}_{\text{min}}+\left[\left({\text{G}}_{\text{max}}-{\text{G}}_{\text{min}}\right)/\left(1+{\text{e}}^{\left(\text{A}\left(\text{V}-{\text{V}}_{\text{o}}\right)\right)}\right)\right]$ where G_ss_/G_i_ corresponds to the normalized conductance at steady state, G_min_ to the residual state, or non-voltage dependent conductance, G_max_ to the maximal conductance, A is a parameter that defines the steepness of the relation and Vo indicates the voltage at which G_ss_/G_i_ = 1/2.

### Single channel analysis

Single channel currents were measured by using freshly split cells, where junctional conductance was low. Unitary junctional currents were digitized during long (1–5 s) voltage pulses of 30–60 mV of both polarities applied to either one of the cells. Amplitudes of unitary opening or closing current events were digitized through a Heka 9000 amplifier (Germany) filtered at 100–500 Hz. Probability distribution histograms of the events and Gaussian distribution best fits for each peak were calculated for each experiment (Origin 7.0). Specific single channels current traces were digitized at 5 kHz and used to generate all point histograms with a bin resolution of 4–8 pS and filtered at 200 Hz.

### Permeability probes

Lucifer yellow (LY; MW: 443 Da; 5% in LiCl 150 mmol/L) or Rhodamine-123 (R-123; MW = 380 Da; 10% in KCl 0.5 mmol/L) were injected iontophoretically in one cell inside a group of cells. The movement of LY or R-123 was detected after 3 min by epifluorescence. Neurobiotin (NB; MW = 287 Da; 20%) was co-injected with R-123 (5% in KCl 0.5 mmol/L). Three minutes after co-injections, cells were fixed with 10% formalin overnight. NB was traced with Avidin-HRP antibodies after permeabilization with 0.25% Tritonx100 in PBS. Molecular modeling of the permeability probes was performed through DS Viewer Pro (Accelrys Inc, CA). Identification of cells forming heterotypic junctions was performed by staining cells with either DiI or Green Cell tracker (Molecular Probes CA), depending on the cells that were going to be injected with fluorescent dyes.

### Asymmetric flux under double whole cell voltage clamp

Asymmetric flux of dye molecules was obtained by using epifluorescence data while total junctional conductance was measured simultaneously in a dual cell voltage clamp configuration. One micropipette used in the clamp was loaded with 5 mM LY while the other was not. Cell pairs were voltage clamped and dye fluorescence inside the cells was measured using a Region of Interest (ROI) from digitized images taken by a cooled camera connected to a computer using Imaging Workbench software (Indec Biosystems, Santa Clara CA) Simultaneously with imaging, the total junctional conductance (Gj) was derived from the measured currents during the application of alternating voltage pulses. Dividing Gj by the reported average unitary channel conductance (γj) yielded the number of functional channels in the junction. The cell receiving the dye directly from the dye-loaded micropipette reached quickly a steady state of fluorescence while the second cell in the voltage clamp slowly received the dye molecules through the gap junction. The slope of change in dye fluorescence over time in the second cell, when the first cell has reached steady state, divided by the number of channels represented the flux of dye molecules per channel.

### Statistical analysis

For all dye transfer experiments, all coupling coefficients or number of molecules calculated from each experimental group were gathered independently. All data were added to an Origin project page and statistical analysis was performed per column to obtain standard error and standard deviation for each experimental group (e.g., all homotypic Cx43). For comparison between experimental groups, we used the same columns to perform a Two Sample *t*-tests with two tails and confidence level of 95%. Histograms for single channel events were generated also through Origin, using Statistical Graphs, and Histogram selections, where the bin size was manually assigned. Best fit for each histogram was performed by the Multiple Peak Fit Tool. Since there were a large number of not very well-defined peaks, we seeded manually the initial mean and standard deviation for each visible peak and then let the program produce the fitting leaving free all parameters. We optimized the fitting through multiple iterations 10–100 until the Chi^∧^2 (goodness of fitting) reached the smallest value and did not change over 10+ iterations.

## Results

**Note:** all connexons' cartoons in **Figures 4**–7 are representing heteromeric combinations that contain three of each connexins in an alternating conformation. This is solely for simplification and visualization purposes. All possible combinations can be inspected in Figure S1.

### Total junctional conductance is reduced in mono-heteromeric channels

**Figure 1:**
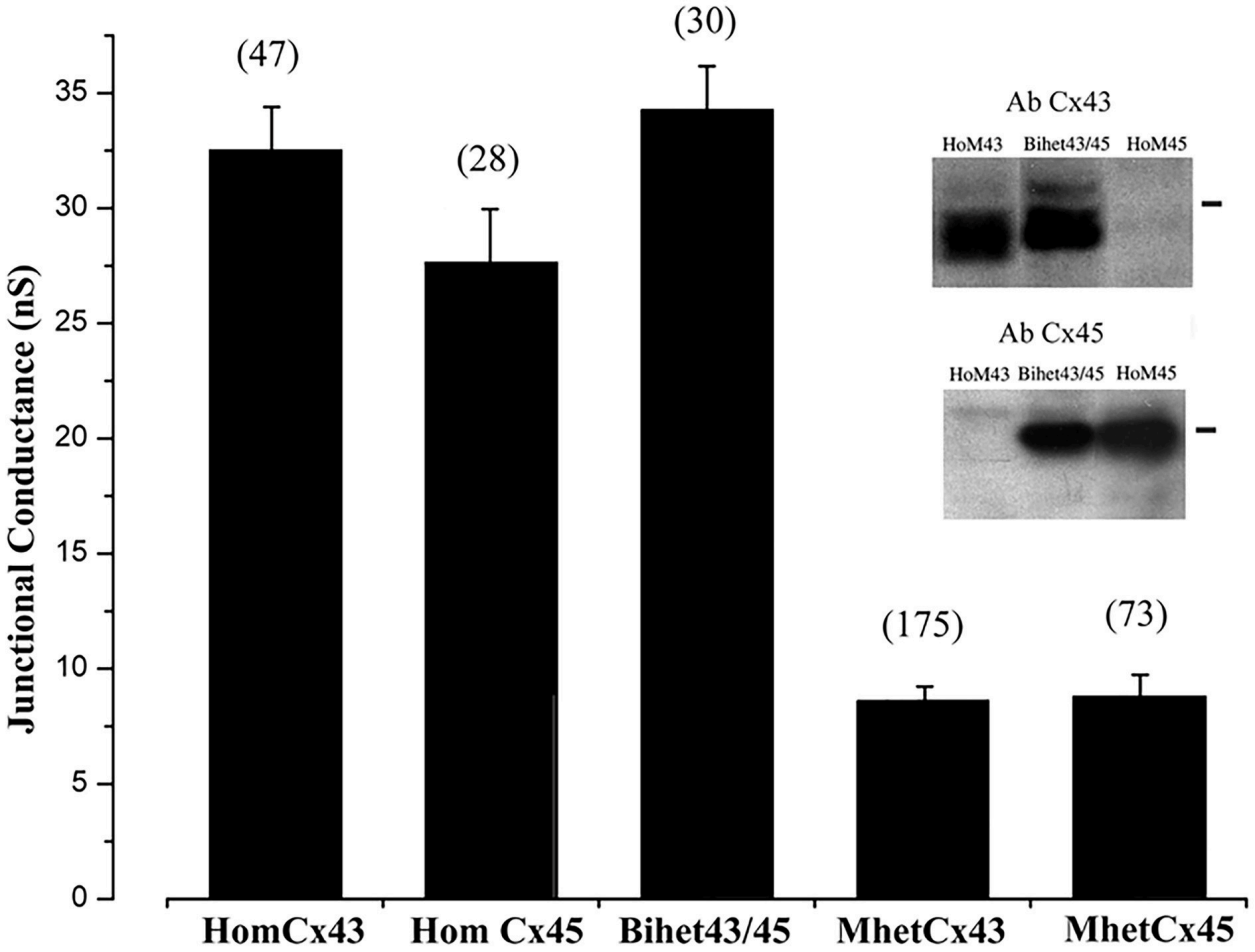
**Junctional conductance measured between HeLa cell pairs expressing distinct homomeric and heteromeric configurations**. Numbers in parenthesis correspond to the cell pairs tested. Inset: Western blot indicating the expression of connexin43 or Cx45 in HomCx43, BiHetCx43/Cx45 and HomCx45 cells. Note that for BihetCx43/45, bands indicating possibly different phosphorylation states of Cx43 are more conspicuous at higher molecular levels.

The first and one of the most striking results was obtained while measuring the total conductance of the different combinations of hetero-oligomeric channels formed from Cx45 and Cx43. As we have previously published, there was no significant difference in total conductance between the cell clones expressing homotypic Cx43, homotypic Cx45, or BihetCx43/45 channels; they exhibited mean total conductances in the range between 27 and 32 nS. However, the total coupling observed for mono-heteromeric channels (MhetCx43 and MhetCx45) was significantly smaller. MhetCx43 and MhetCx45 had total junctional conductances of 8.59 ± 0.64 nS (*n* = 175) and 8.37 ± 0.99 nS (*n* = 73) respectively (Figure 1). This was all the more surprising, since we had predicted from the theoretical distribution of channels that these conductances should be at least 24 and 31 nS (see Discussion below and Figure S1).

To determine protein expression in these cells, we performed Western blots of total protein extracts prepared from cells 2 days in culture expressing connexins in distinct configurations. As presented in Figure 1 (inset), Cx43 was detected in cells forming HomCx43 and BihetCx43/Cx45 channels, but not in HomCx45 cells. Interestingly, electrophoretic mobility of the Cx43 forms were not identical in HomCx43 and in BiHetCx43/Cx45 cell homogenates. In the BiHetCx43/Cx45 cells, Cx43 contained a greater proportion of slow mobility forms, consistent with increased phosphorylation of the connexin protein (Figure 1 Inset, second lane). Although we did not detect Cx45 from cells expressing only Cx43 (HomoCx43), we cannot exclude some expression of endogenous Cx45 in HeLa cells, because in our previous work we detected few levels of Cx45 protein from highly confluent HeLa cell cultures after concentration by alkali extraction (Elenes et al., 2001) indicating that this cell line can endogenously express this protein.

### Heteromeric connexons reduce the distribution of unitary conductances

**Figure 2:**
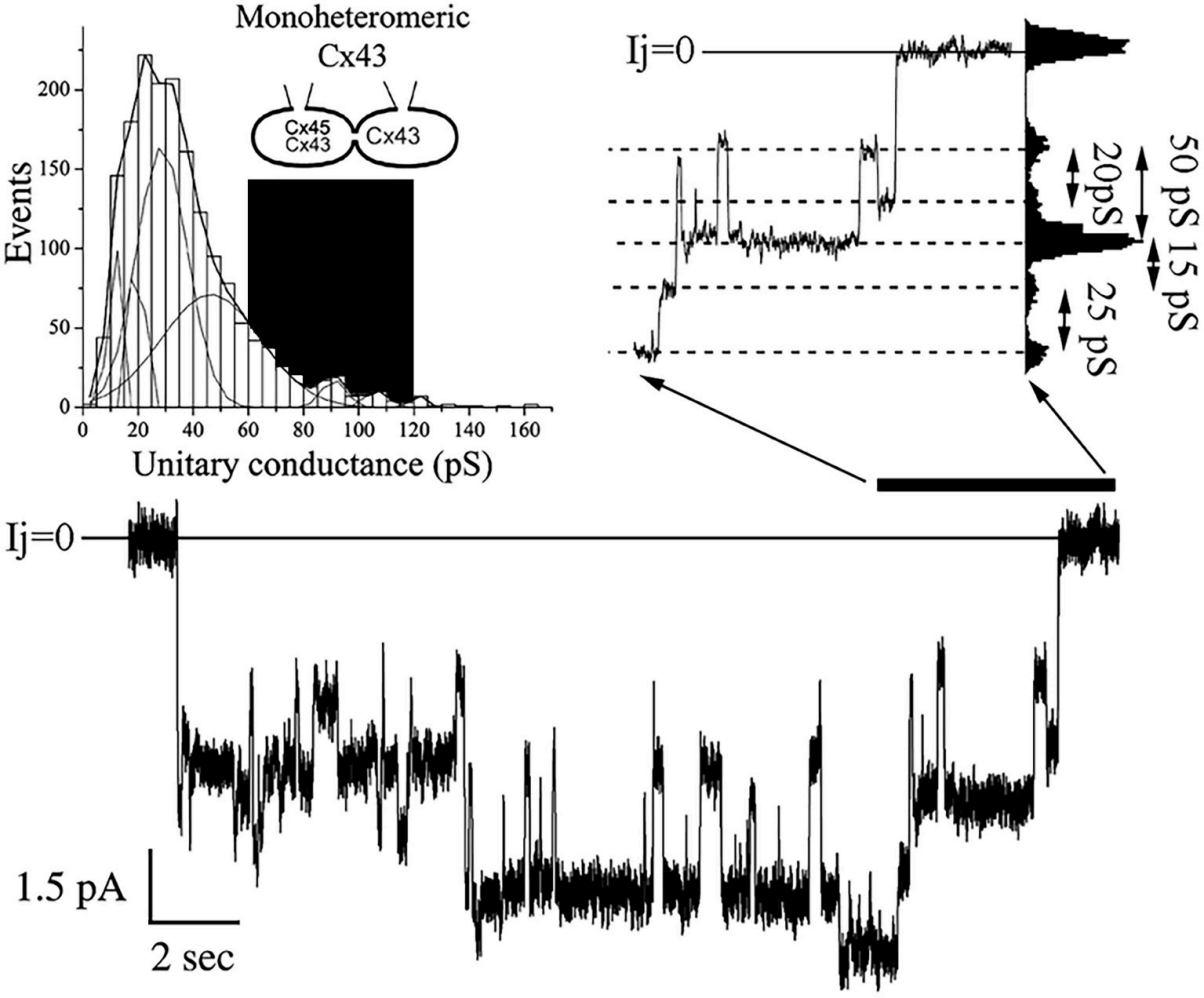
**Unitary conductance distribution obtained from mono-heteromeric Cx43 channels**. The histogram on the top left represents 1,689 events where 9 different Gaussian functions were needed to obtain the best fit to the distribution. The mean value for all transitions was 36.8 ± 0.54 pS. The black area represents the theoretical values of conductances expected. Note that most of the unitary conductances are smaller than the predicted values. The junctional current trace at the bottom is an example where multiple channel transitions could be observed. This trace was obtained during a depolarizing 60 mV pulse applied to the cell expressing Cx43. Notice that there is little decline in the total current indicating low voltage dependence. The current region indicated by the black bar at the right of the trace was expanded on time to resolve various transition sizes. The values of conductance for these transitions were obtained through all-point histograms, as the one shown on the right of the expanded trace.

As a second approach to study the properties of mono-heteromeric channels, we performed studies with cell pairs where junctional conductance was low enough to record unitary current events without the application of uncoupling agents. In cell pairs expressing MhetCx43 channels, we detected unitary currents that corresponded to conductances from 20 to 150 pS. These conductances were calculated from current traces like the one presented in Figure 2. In this particular trace, obtained during a transjunctional voltage pulse of 60 mV, multiple conductive states were reached through transitions of distinct magnitudes. The difference in magnitude was more clearly calculated using an all-points histogram from the ionic current section expanded and digitized (Figure 2 top right), and transitions of 15, 20, 25, and 50 pS were detected. The frequency of these unitary current transients or events calculated from 8 different cells and 1,689 transitions corresponded to conductance values of 20–30 pS (Figure 2 top left). The distribution covered a wide range of conductances and was fitted with the sum of 9 Gaussian distributions where the mean of all events corresponded to γ_j_ = 36.8 ± 0.54 pS; a major peak was detected at 22.5 pS and smaller peaks at 12.5, 17.5, 27.5, 47.5, 92.5, 97.5, 107.5, and 125.5 pS.

**Figure 3:**
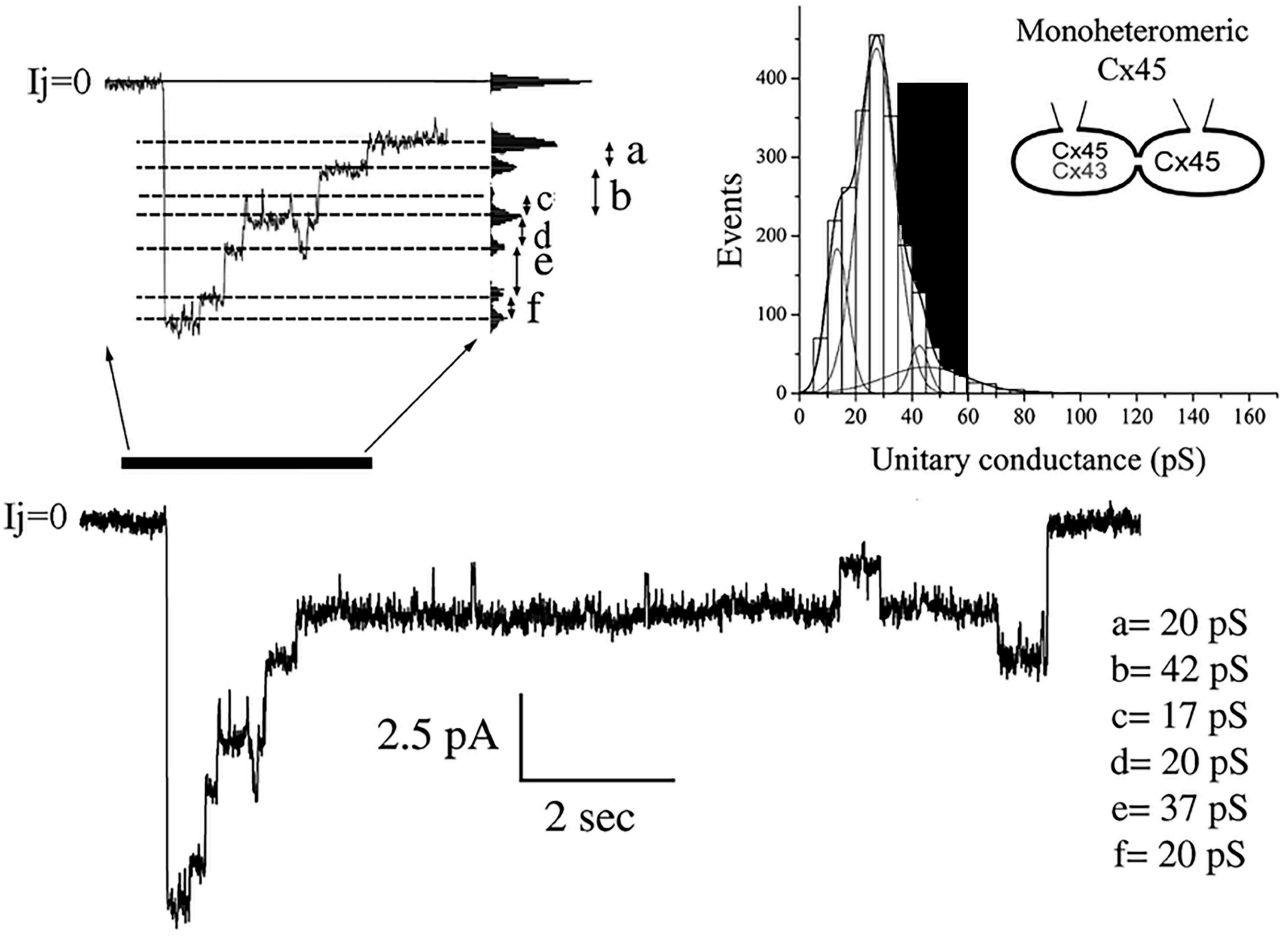
**Unitary conductances distribution obtained from mono-heteromeric Cx45 channels**. The histogram on the top right represents 2,182 events where 4 different Gaussian functions were needed to obtain the best fit to the distribution. The mean value for all transitions was 27.84 ± 0.25 pS. The gray area represents the theoretical values of conductances expected. Note that most of the unitary conductances are smaller than the predicted values. The junctional current trace at the bottom is an example where multiple channel transitions could be observed. This trace was obtained during a depolarizing 60 mV pulse applied to the cell expressing Cx45. Notice that there is strong decline in the total current indicating high voltage dependence. The current region indicated by the black bar at the left of the trace was expanded on time to resolve various transition sizes. The values of conductance for these transitions were obtained through all-point histograms, as the one shown on the right of the expanded trace.

Current transitions from cells expressing MhetCx45 channels were also obtained from poorly coupled cells where single unitary currents could be detected without the use of uncoupling agents. These conductances were calculated from current traces obtained under voltages ranging from 30 to 60 mV at both polarities. In the example obtained during a transjunctional voltage pulse of 60 mV is presented in Figure 3, multiple conductive states were reached through transitions of distinct magnitudes. The difference in magnitude was more clearly calculated using an all-points histogram from the expanded current region (Figure 3 top left); transitions corresponding to conductance values of 17–42 pS were detected. The frequency of such unitary current transients or events calculated from eight different cells pairs and 2,182 transitions corresponded to conductance values between 20 and 50 pS; this distribution is presented as an events histogram in Figure 3 (top right). The distribution covered a wide range of conductances and was fitted with the sum of 4 Gaussian main distributions where the mean of all events corresponded to γ_j_ = 27.8 ± 0.3 pS. The major peak corresponded to a mean of 27.5 pS with the other peaks falling at 12.5, 42.5, and 45 pS. The theoretical distribution of conductances had been expected to be in the dark region of the plot with conductances between 30 (homotypic Cx45) and 60 pS (heterotypic Cx45-Cx43).

### Heteromeric connexons reduce the voltage dependence of homomeric connexons

**Figure 4:**
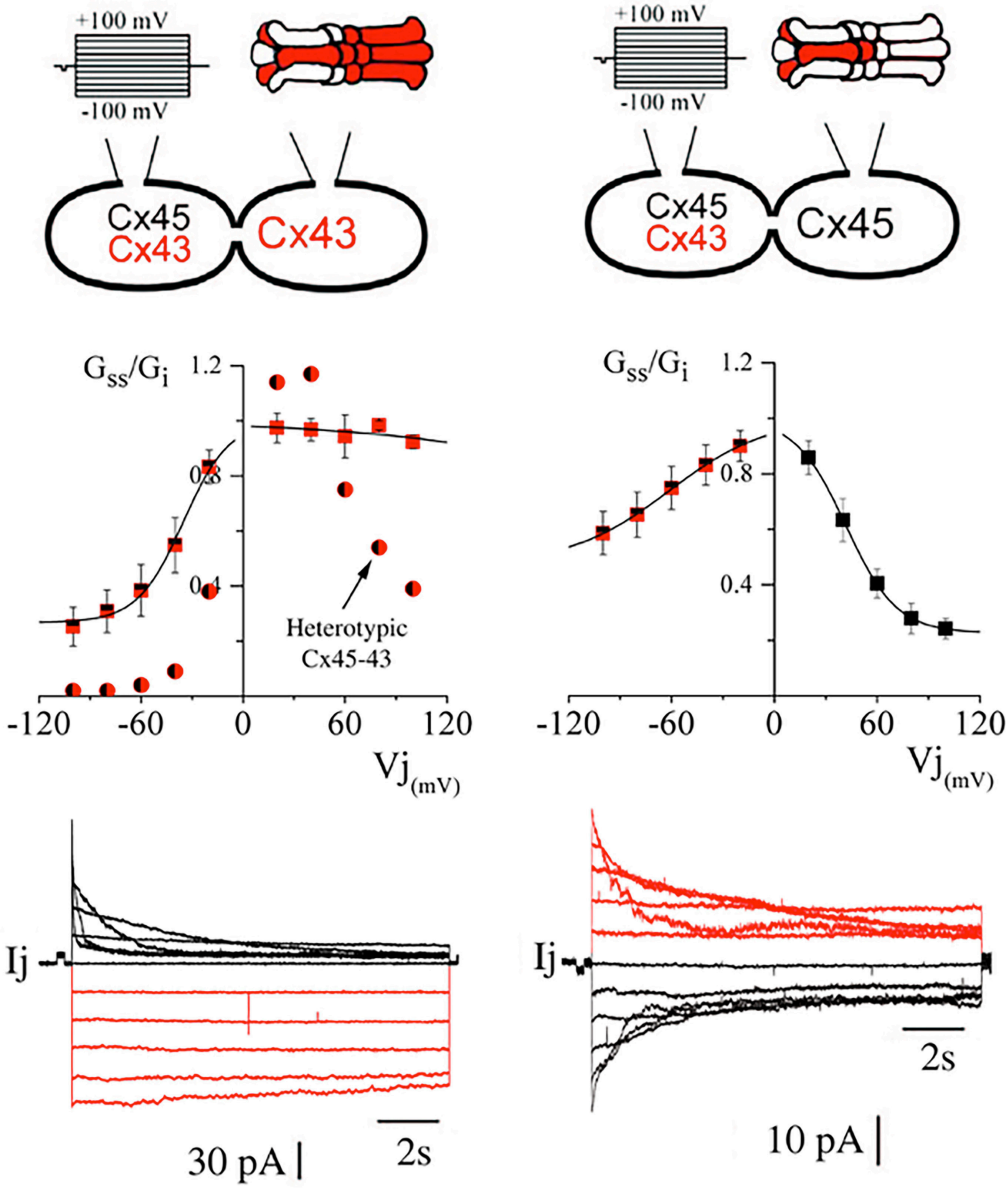
**Comparison of voltage dependence from hetero-multimeric channels**. In both panels, we present a representation of the connexin combination **(top)** indicating the cell that was stimulated, the average of steady state conductance levels **(middle)** and representative current traces where the color matches the connexon where Cx43 is more abundant **(bottom)**. As reported before, in heterotypic channels, voltage gating of Cx43 homomeric connexons appears to be inhibited. On the left, heterotypic channels formed by Cx43 with Cx45 connexons induce a reduction in the gating kinetics of Cx43 (circles). This gating inhibition is even stronger when the opposing connexons are heteromeric (squares). On the right, heteromeric connexons form channels with HomCx45 connexons (One heteromeric and the other homomeric Cx45) that appear to be less rectifying to voltage gating.

As we have previously reported (Elenes et al., 2001; Bukauskas et al., 2002), the heterotypic combination of connexons formed by Cx45 and Cx43 yield channels where Cx45 connexons gate with similar kinetics to those found in Cx45 homotypic channels, whereas Cx43 connexons gate substantially slower and with less voltage dependence (Figure 4 left panel). We have now found that when heteromeric connexons are forced to couple with homomeric Cx43 connexons, the resulting voltage dependence becomes even more asymmetric. The voltage sensitivity of the heteromeric connexons appeared to be a mixture between the sensitivities of HomCx43 and HomCx45 connexons with Vo at −35.09 ± 1.96 mV (*n* = 6; see Table 1), although the residual conductance (0.27) was substantially larger than in homotypic Cx45 channels (Figure 4 left panel; 0.14 ± 0.01 see Table 1). In contrast, the voltage gating for Cx43 appeared to have been completely abolished, although some gating was observed. (Gating parameters are shown in Table 1). The voltage gating for MhetCx45 channels showed in average less asymmetry, where the voltage sensitivity for Cx45 homomeric connexons was reduced to a *Vo* = 41.7 ± 0.34 mV and its residual conductance corresponded to 0.23, closer to HomCx45. Here, the gating of the heteromeric connexons appeared to be a mixture between the gating of Cx43 and Cx45.

**Table 1:** **Transjunctional voltage gating properties of Homotypic, Bi-heteromeric, and Mono-heteromeric gap junction channels formed by Cx43 and Cx45**.

	**Vo**_**(mV)**_			**G_max_**	**G**_**min**_	
	**N**	**Negative**	**Positive**		**Negative**	**Positive**
HomCx43	4	−60.15 ± 0.68	68.40 ± 0.38	0.98	0.32 ± 0.01	0.31 ± 0.01
HomCx45	5	−17.44 ± 0.30	21.79 ± 0.56	1.5	0.14 ± 0.01	0.16 ± 0.01
		**Heteromeric connexons**	**Homomeric connexons**		**Heteromeric connexons**	**Homomeric connexons**
MhetCx43	6	−35.09 ± 1.96	322.83 ± 202.38	1.00	0.27 ± 0.02	0^*^
MhetCx45	10	−61.42 ± 2.53	41.78 ± 0.34	1.00	0.48 ± 0.02	0.23 ± 0.005
	0					

* *forced to obtain Boltzmann fitting*.

### Heteromeric connexons reduce the permeability to LY and rhodamine123

**Figure 5:**
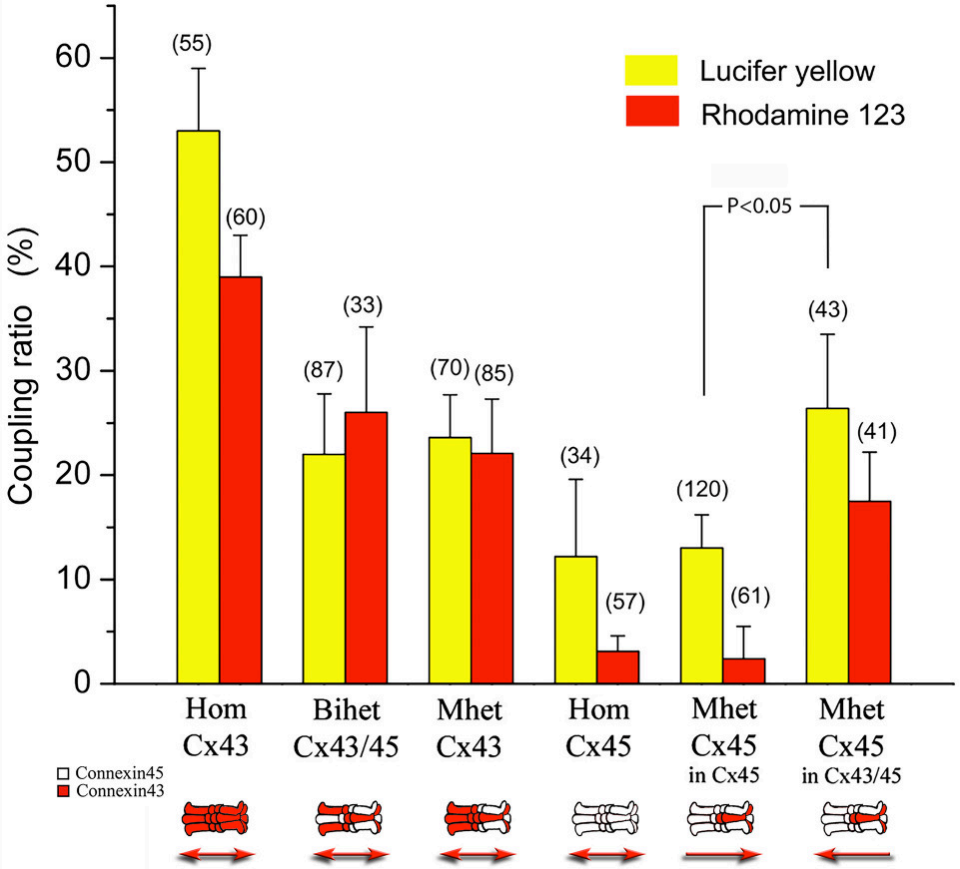
**Coupling coefficients calculated in homotypic, bi-heteromeric and mono-heteromeric gap junction channels formed by Cx43 and Cx45 after Lucifer yellow (LY) or Rhodamine 123 (R-123) was injected into the cells**. In general, the coupling coefficients after LY injections or Rhodamine were reduced in all connexin combinations compared to HomCx43 channels. In particular, LY permeability resulted significantly higher compared to that of R-123 in those pairs where the injection occurred at the side where Cx45 was present or more abundant (e.g. for HomCx45, MhetCx45, and MhetCx43 injected at Cx43/Cx45 side). Interestingly, there is a significant asymmetry observed in MhetCx45 channels, where permeability is higher for both dyes if the injection is performed at the side where heteromeric channels are formed. Numbers on top of the columns indicate the number of experiments/number of injections.

Two dyes of similar dimensions and 3D shape (see Table 2 and Figure S2) but distinct electrical charges were tested: Lucifer Yellow and Rhodamine123. The highest coupling ratio for both of the dyes was observed in cells expressing homotypic Cx43 channels (likely due to the low charge selectivity reported for these channels)^20^. As shown in Figure 5, the coupling coefficient (defined in Methods) for LY reached 52%. For R-123, this coefficient was smaller (40%) but in our conditions, its fluorescent intensity was lower than LY.

**Table 2:** **Characteristics of fluorescent probes used**.

	**Charge**	**Molecular weight, g/mol**	**Dimensions, A°**
Lucifer yellow	–2	443	12.2 × 11.2 × 6.1
Rhodamine 123	+2	380	11 × 11.4 × 6.9
Neurobiotin	+1	287	17.1 × 0.2 × 0.3

The permeability of LY and R-123 through BihetCx43 and MhetCx43 channels was significantly reduced to values close to 25% (Figure 5, second and third pairs of bars). There was no significant difference between LY and R-123 coupling coefficients for either connexin combination. According to injections performed in HomCx45 channels, the coupling coefficient for LY was also smaller (27%) than that determined in HomCx43 channels and was even smaller for MhetCx45 channels. Interestingly, the coupling coefficient for R-123 was drastically smaller in the homotypic Cx45 channels, reaching only 4% (Figure 5 compare 4th and 5th pairs of bars).

One important property that appeared in the mono-heteromeric channels is that the coupling coefficient in MhetCx45 channels depended on the side of the junction where the dye was injected. In this case, the injection of LY and R-123 at the heteromeric connexon side yielded a coupling coefficient twice as great and with apparently less charge selectivity (Sixth column pair Figure 5).

### Differences in directional flux across mono-heteromeric channels formed by Cx45 and Cx43 are related to size and not charge

**Figure 6:**
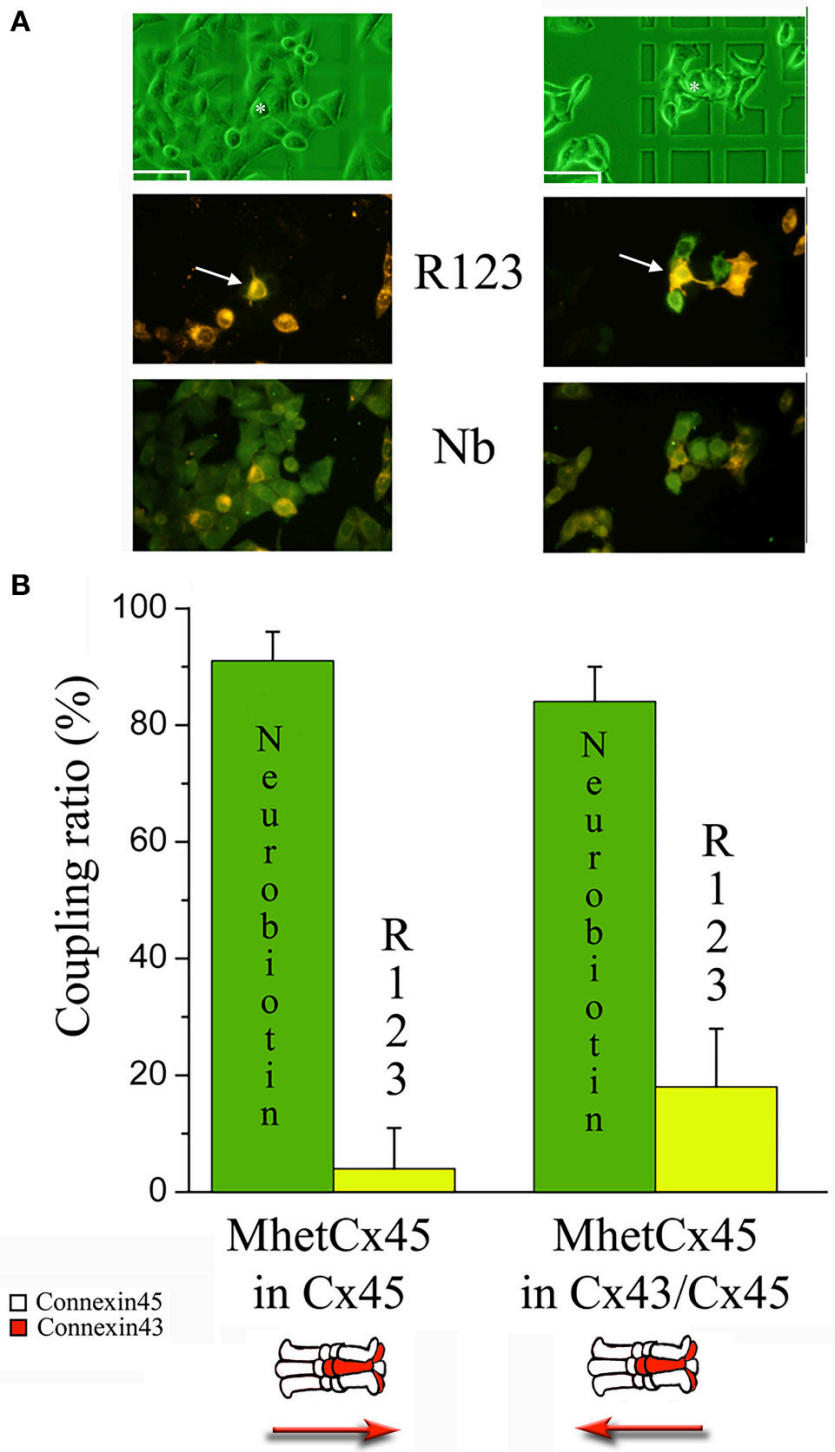
**Permeability of Rhodamine123 and Neurobiotin through MhetCx45 channels**. **(A)** Sequence of micrographs indicate typical results after co-injection of R-123 and NB. Left panel corresponds to a co-injection of R-123 and NB in Cx45 expressing cells (white arrow). The right panel corresponds to an injection in the heteromeric Cx43/Cx45 side (white channel). Note that in both cases, Neurobiotin (dark green; photos at the bottom) permeates to most of the surrounding cells (light green at the bottom micrographs), independently of the side of injection. In contrast, R-123 permeates faster when injected at the heteromeric side (green at the middle micrographs). Calibration marks are 50 μm. **(B)** Coupling ratio calculated for co-injections performed into cells expressing Cx45 only (left columns) or co-expressing Cx43/Cx45 (right columns).

To determine the molecular basis of low permeability and the differences in directional flux across mono-heteromeric channels, we performed experiments co-injecting a smaller and positively charged molecule (NB) with R-123 and then compared its flux with that of R-123 (Figure 6). During this new set of experiments, coupling ratio for R-123 injected into Cx45 side was 34.67 ± 3.32;19 (Mean ± SE; n) and that of Cx45/43 was 42.05 ± 4.96;19 with a *p* = 0.20. Coupling ratio for NB injected into Cx45 side was 62.87 ± 6.66;23 and that of Cx45/43 was 66.64 ± 5.52;19 with *p* = 0.67. Both R-123 and NB showed permeability across mono-heteromeric channels, with a larger flux in the direction Cx43/45 → Cx45. NB showed twice the diffusion compared to R-123 suggesting that heteromeric connexons are capable of inducing some rectification to large molecules like R-123 where size appears to be more important than charge.

**Figure 7:**
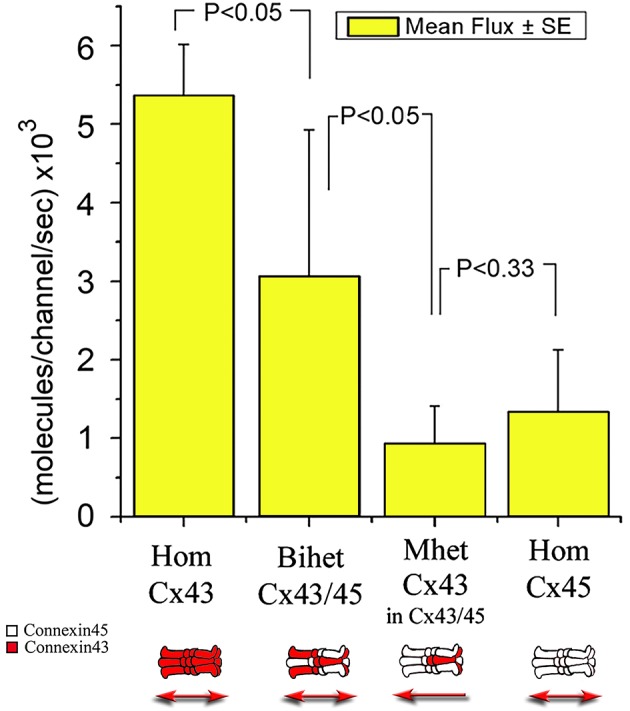
**Graph comparing mean unidirectional flux of LY per single channel among different channels made from homomeric, mono-heteromeric and bi-heteromeric combinations**. For MhetCx43, the pipette with dye was on the heteromeric side of the junction (Cx43/Cx45).

### MhetCx43 channels have very low unidirectional flux

To determine with more accuracy the flux of molecules across heteromeric channels, we simultaneously recorded the junctional conductance in a cell pair and the movement of fluorescent Lucifer yellow across the junction (Valiunas et al., 2002). With this method, we found that among homotypic, bi-heteromeric and mono-heteromeric arrays of Cx43 and Cx45, mono-heteromeric channels were the ones to allow the lowest flux of LY, in this case in the heteromeric to homomeric direction.

HomCx43 channels allowed a large LY flux at 5.37 ± 0.65 × 10^3^ molecules per channel per second (m/c/s; *n* = 5), while HomCx45 channels allowed only 1.35 ± 0.78 × 10^3^ m/c/s (*n* = 5). BihetCx43/45 channel flux was 3.07 ± 1.86 × 10^3^ m/c/s (*n* = 5), halfway between the homomeric-homotypic channels.

MhetCx43 channels allowed less flux of LY molecules compared to HomCx43, HomCx45 and Bihet43/45 channels. Moreover, flux had a tendency to flow less from the Cx43/45 to Cx43 (0.55 ± 0.39 × 10^3^ m/c/s; *n* = 5) compared to fluxes measured from Cx43 toward Cx43/45 (0.94 ± 0.47 × 10^3^ m/c/s *n* = 5) although this difference was not significantly different.

## Discussion

In this manuscript, we have shown that the co-expression of Cx45 and Cx43 induces the formation of mono-heteromeric channels with properties different than the ones recorded from their parental homomeric counterparts, including reduced unitary conductance, reduced permeability to large dyes and reduced transjunctional voltage dependence. Moreover, our data strongly suggest that this combination of connexins could be an effective physiological method to regulate the levels of communication between cells and in tissues with multiple cell types expressing more than one connexin type (e.g., Neurons, oligodendrocytes and astrocytes in brain, or fibroblasts, myocytes and SA node cells in heart), their membrane distribution and permeability can establish unique communication pathways among these cells, controlling the flow of information between them. In short, the mono-heteromeric configuration confers a smaller unitary conductance and a stronger asymmetry upon the channels reflected by a robust voltage dependence rectification and by an asymmetric flux to large fluorescent dyes. A recently published computational model has reproduced the molecular asymmetry in heterotypic channels and stated the influence of pore shape and electrostatic interactions in the process (Mondal et al., 2017).

The unitary conductance of mono-heteromeric channels, formed by pairing with either Cx43 or Cx45 homomeric connexons was significantly smaller than predicted by the hypothesis that the limits of unitary conductance should be determined by the expression levels of Cx43 or Cx45 in the heteromeric side of the channel. According to this hypothesis, the largest unitary conductance in a junction is expected to correspond to channels where Cx43 is present (Cx43 only channels on the far-left side in Figure S1B) and the smallest when only Cx45 is present (Cx43 absent), forming a pure homotypic-homomeric channel (channels on the far right in Figure S1B). For those combinations located in between, we initially proposed that substitution of a Cx45 subunit for Cx43 (in the heteromeric side) would reduce the channel unitary conductance by 1/6th of the difference between the unitary conductance of a HomCx43 channel (120 pS; no substitution) and a HetCx43/45 channel (60 pS; all connexins substituted). These values are presented in the table inserted in Figure S1A. The substitution of 2, 3, or 4 subunits may occur in three different arrays as depicted in Figure S1B. The heteromeric connexon (on top of each channel) could have up to 13 distinct conformations although the properties of the three central groups might be difficult to distinguish. In 2002, we have published data showing the presence of small conductances in bi-heteromeric channels (Martinez et al., 2002), and we theorized that the intermediate combinations would yield intermediate conductances between 120 (HomCx43) and 60 pS (HetCx43/45) (Elenes et al., 2001). However, according to our data presented in Figures 2, 3, this was not the case for either of the mono-heteromeric channel combinations which had mean γj values of 27 and 37 pS respectively. One way to interpret this is that the docking of heteromeric and homomeric connexons induces a change in the channel structure (docking induced transformation). Another possible explanation for these reductions in conductance is an altered phosphorylation of Cx43, as suggested by Figure 1. The interaction between Cx45 and Cx43 might change conformations in the proteins making them more susceptible phosphorylation leading to smaller conductive heteromeric channels. Intra-connexon inter-connexin domain interactions (e.g., Cx43-Cx45 NT-NT or CT-CT interaction could also explain these effects) since Cx domain interactions have been reported to alter channel function (Shao et al., 2012). In addition, we have found that co-expression of Cx43 truncated at its C-terminus domain with Cx45 form gap junctions well-permeable to Lucifer Yellow, by contrary, the heteromeric channels formed by full length Cx43 and Cx45 present restricted permeability to LY (Martinez et al., 2003), suggesting that possible inter-subunit interaction between Cx43 CT domain and Cx45 could modulate GJ channel function. To determine the mechanism involved for either docking or phosphorylation will require further study. Suffice at this point is to say that the vast majority of functional channels (>80%), in an *in vitro* induced mono-heteromeric junction, show unitary conductances below 45 pS. These small unitary conductance events have already been observed in BihetCx43/45 channels (Martinez et al., 2002) and are consistent with the observation that the co-expression of connexins yielded smaller channels, since the total distribution from bi-heteromeric channels also shifted to smaller conductances than those predicted.

Linked to the low conductance of the mono-heteromeric channels we observed a strong reduction in the permeability to Lucifer yellow for heteromeric channels. As shown in Figure 3, the coupling coefficient for Homotypic Cx43 channels is high, but it becomes reduced to 22% in BihetCx43/45 channels. This low permeability was reported in our previous work (Martinez et al., 2002). The coupling coefficient reduction could be explained by the reduction in effective permeability to large molecules, therefore, the addition of Cx45 subunits to the channels resulted in a lower permeability to both LY and R-123. For MhetCx45 channels, the permeability for LY and R-123 was strongly reduced into coupling levels of 12 and 4% respectively. This indicates that these channels were able to strongly select small molecules. Interestingly, our permeability experiments indicate that HomCx43 channels may be somewhat anion selective, since LY (*z* = 2e^−^) crossed the junctions with higher efficiency than R-123 (*z* = 1e^+^). Cx45 homotypic channels have been reported to be cation selective; hence the difference that we obtained might be dominated not by the charge but by the shape of R-123. Although the two molecules (LY and R-123) have similar molecular weights, the structure of R-123 appears bulkier, as shown in Figure S2. Supporting the interpretation that the selectivity of these channels is based on the size but not the charge of the molecules, we have observed that Neurobiotin crossed readily all combinations of connexins, and this can be explained because it is a smaller molecule(Martinez et al., 2002). At any rate, the combination of Cx45 homomeric connexons with heteromeric connexons rendered channels highly selective for a molecule size in the range between 300 and 400 Da. The most interesting result from these permeability data is that depending on the side of the junction injected, the permeability to both, LY and R-123 was significantly different. Lucifer Yellow and R-123 permeated with a coupling coefficient which was three times larger if injected from the heteromeric side. No significant difference was recorded for MhetCx43. This indicates that asymmetries in flux ratio could be established depending on the type of connexon expressed. Measurements using simultaneous voltage clamp and permeability data had a limitation for small conductive channels and, it was not possible to determine differences in fluxes in cell pairs expressing MhetCx43 or MhetCx45 channels. The statistical errors calculated for our fluxes surpassed the fluxes themselves. Besides, differences in cell size or membrane leak prevented obtaining significant data. Nonetheless, injection experiment results accord with our *in vitro* and *in silico* experiments where asymmetric fluxes have been demonstrated using Brownian dynamics (Mondal et al., 2017). These models point to the distribution pattern of charged particles near the pore's mouth as the major cause of the observed asymmetric flux (Mondal et al., 2017). These mechanisms may also explain the observations from experiments performed on neuronal-glial coupling where a directional flux of molecules was reported (Robinson et al., 1993). Moreover, our results after co-injection of NB and R-123 indicate that the directional selectivity is mostly based upon size, since the flux of NB was independent of the side injected (Kanaporis et al., 2011).

The small unitary conductance of MhetCx45 and MhetCx43 channels clearly impacted in the total conductance between cells. In control conditions the total conductance of mono-heteromeric channels is ~30% of the junctions formed by homotypic or BihetCx43/45 channels. According to the unitary conductance distribution presented in Figure 1 (mean of 37 pS), there should be approx. 243 functional channels present in these MhetCx43 junctions (9,000 pS/37 pS per channel). If we consider that in average homotypic Cx43 cells express 266 channels (36,000 pS/120 pS per channel), it appears as if the maximal number of channels (limited by the expression of Cx43 cells) was reached. This suggests that most of the conductance predicted in our initial hypothesis were substantially larger, since the product of 250 channels with conductance between 60 and 120 pS at the probability suggested in Figure 6A when Cx45 and Cx43 are expressed at 50% will result in a total conductance of 27 nS. It remains to be determined which combination of heteromeric connexon becomes favored. At this point we may exclude homomeric Cx43 and Cx45 connexons because they represent only 5 and 2% in the general distribution of conductance (see Figure 1). It is worth also mentioning that the higher coupling ratio and LY flux for BihetCx43/Cx45 combination, relative to either mono-heteromeric combinations suggest that there is a preference for the formation of homotypic-homomeric gap junctions.

Another possibility to explain the small total conductance in MhetCx45 channels would be the difference in expression of both connexins. In the worst-case scenario where co-transfected cells we expressing mostly Cx45, due in part to the expression of endogenous Cx45, the average channel conductance should be close to 30 pS (as in HomCx45 channels), but the distribution of conductances measured was significantly smaller.

In the same fashion as MhetCx43 channels, the impact of small unitary conductance of MhetCx45 channels yields a strong reduction in the total conductance of the junctions. In this case, the maximal number of channels will be determined by the expression of Cx45 homomeric connexon, since it is predicted that there will be an abundance of connexins in the heteromeric side. According to the main unitary conductance distribution recorded from MhetCx45 expressing cells (Figure 2) their junctions should have in average ~300 channels (9,000 pS/27 pS per channel). This number is about half of the number of channels in control HomCx45 cells, which should have ~600 functional channels (27000 pS/45 pS per channel). This does not correspond to the total conductance predicted in our initial hypothesis, since for 300 channels at the predicted probabilities shown in Figure 5A, the MhetCx45 channels should yield a total conductance of ~30 nS.

Hence, we can conclude that the mechanism of reduction of connexon conductance was not all through differential selection between connexons, since apparently, most of all the available connexins were part of functional channels. According to our data, either the interaction of Cx45 and Cx43 in a connexon and/or the docking between heteromeric connexons yields full channels with lower conductivity or non-conductive at all. The mechanism has not yet been determined, but can include also differences in affinity of the different connexins interacting.

The presence of heterotypic channels has been reported as a way to produce channels that gate asymmetrically to transjunctional voltage. This has been reported for various connexin combinations(White et al., 1995; Elenes et al., 2001; Bukauskas et al., 2002) expressed in oocytes and mammalian cells. For Cx43-Cx45 heterotypic junctions we have reported that there is strong reduction in the gating kinetics of Cx43 connexons and that there is a robust decrease in g_min_ from both sides of the junctions. This effect has been proposed to be due to the difference in resistances of each connexon in a channel. Hence, the voltage drop across the junction channel will not occur proportionally across both connexons, but it will be stronger for the one with smallest conductance (Bukauskas et al., 2002). It appears that our data supports this proposed mechanism. When MhetCx43 channels are formed, the voltage dependence of the monomeric connexon appears to be lost and just a small gating can be recorded. This indicates that the heteromeric connexons not only have a smaller conductance compared with that of Cx43 homomeric connexons but it is even smaller than HomCx45 connexons. This is strongly supported by our single channel conductance data where the main unitary conductance corresponds to 37 pS. To support even further these data, when MhetCx45 channels are formed, the voltage gating asymmetry becomes strongly reduced, as shown in Figure 5. In this case, a smaller conductive connexon (HomCx45) faces the heteromeric connexons producing channels where the voltage drop across each connexon is no different than in heterotypic or MhetCx43 channels. Therefore, the voltage asymmetry becomes reduced while directional fluxes appear.

In summary, according to the unitary conductance, voltage dependence and permeability of mono-heteromeric channels, those heteromeric Cx45 and Cx43 connexons that participate in functional mono-heteromeric channels have a small unitary conductance and when paired with homomeric Cx45 or Cx43 connexons provide the junctions with a strong voltage dependent and dye flux asymmetry and flux rates depending on the channel orientation.

The presence of functional heterotypic and/or mono-heteromeric channels in cardiac myocytes, or in other cells that express two or more different connexins are expected to enable, not only conductive steady state differences, but also an alteration in the gating responsiveness and permeability differences among the cells. Functionally, the formation of MhetCx45 or MhetCx43 channels may represent a significant barrier for the movement of current or metabolites across a tissue. This could be physiologically related to the distribution of connexins in the SA node where Cx43 is expressed mostly at the musculature, and the inside of the SA node or AV node co-expresses other connexins including Cx43 and Cx45. At the SA node, not only the structural separation between the tissues but also the expression of connexins could represent a barrier that allows enough isolation for the SA node to maintain its functional integrity as pacemaker without being subdued by the electrotonic hyperpolarizing currents from the musculature, but at the same time will enable enough communication to allow the continuation of an action potential.

## Author contributions

All the authors participated in the thorough review of the manuscript. We all have accepted in participating in the publication of this scientific original report. GZ, NA, DA, VH, OA, and APM contributed with original experiments and data analysis as well with the revising of the intellectual content of the manuscript. ADM and EB developed the initial cell clones that have been used through the experiments. ADM, EB and APM generated the initial concept of the manuscript and contributed to the initial draft. All have agreed on being accountable for all aspects of the work involved in this manuscript.

### Conflict of interest statement

The authors declare that the research was conducted in the absence of any commercial or financial relationships that could be construed as a potential conflict of interest.

## Abbreviations

Cx connexin: MhetCx43 Oligomultimeric channels (mono-heteromeric) where one connexon is homomeric Cx43
MhetCx45: Oligomultimeric channels (mono-heteromeric) where one connexon is homomeric Cx45
HomCx45: Oligomonomeric channels (homotypic) formed of Cx45
HomCx43: Oligomonomeric channels (homotypic) formed of Cx43
HetCx43/45: Oligomultimeric channels where each connexon is homomeric Cx45 or Cx43
BihetCx43/45: Oligomultimeric channels (bi-heteromeric) where both connexons are formed of Cx43 and Cx45.
