# Supplementary material for: Mono-Heteromeric Configurations of Gap Junction Channels Formed by Connexin43 and Connexin45 Reduce Unitary Conductance and Determine both Voltage Gating and Metabolic Flux Asymmetry
Source: Front Physiol. 2017 May 29;8:346. doi: 10.3389/fphys.2017.00346 (PMC5447054; doi:10.3389/fphys.2017.00346)
Supplement: Supplementary file 1 [file DataSheet1.DOCX]

**Supplemental Material**


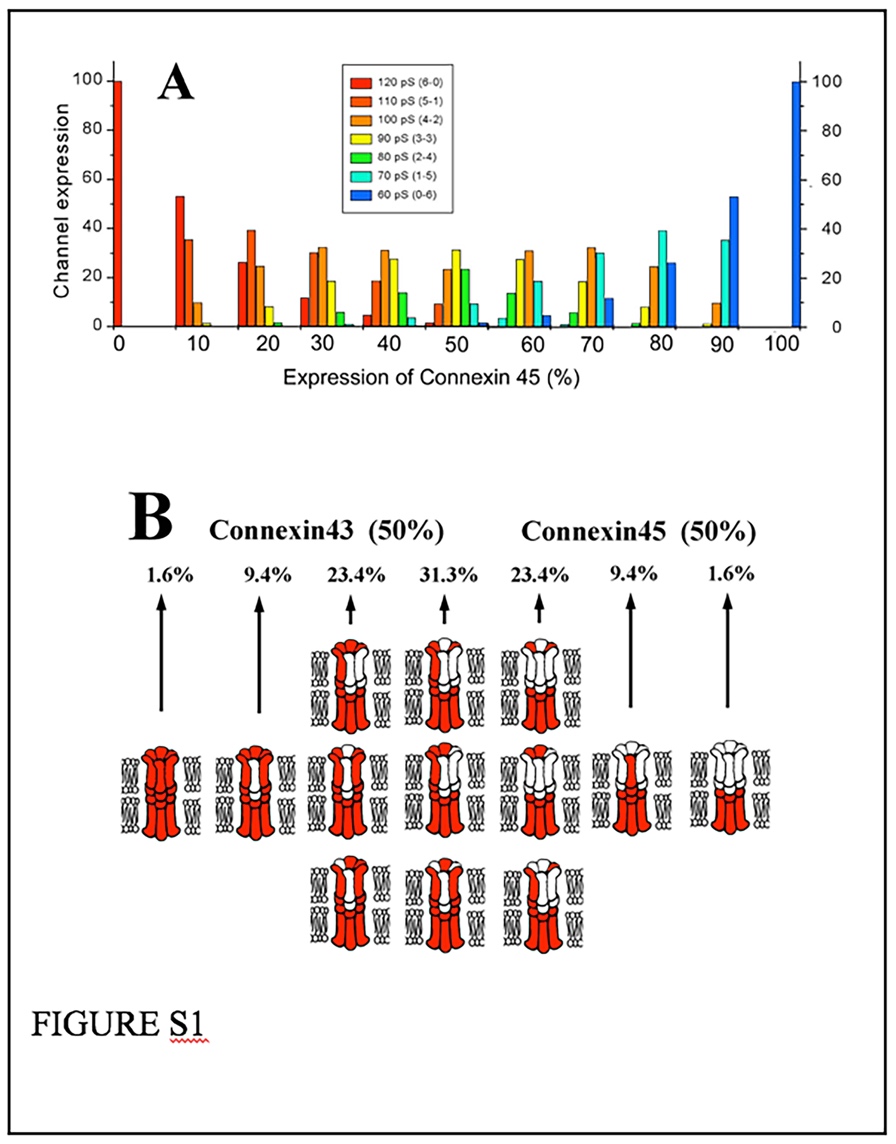


**Figure S1.** Theoretical conductances and combinations of connexin43 and Cx45 in mono-heteromeric Cx43 channels. A. Histogram representing the predicted distribution of unitary conductances of mono-heteromeric channels as a function of the expression ratio between Cx43 and Cx45. If Cx45 is not expressed in a cell where heteromeric channels are supposed to form, then 100% of channels are expected to be homotypic Cx43 (Red far left) with γj=120 pS. If Cx43 is not expressed, then 100% of the channels will be heterotypic (Blue, far right) with γj= 60 pS. In between the different colors indicate the distinct unitary conductances. B. Schematic representation of the distinct possible combinations of mono-heteromeric channels. The percentages indicated correspond to a cell pair where the co-transfected cell expresses equal amounts of Cx43 and Cx45.

**
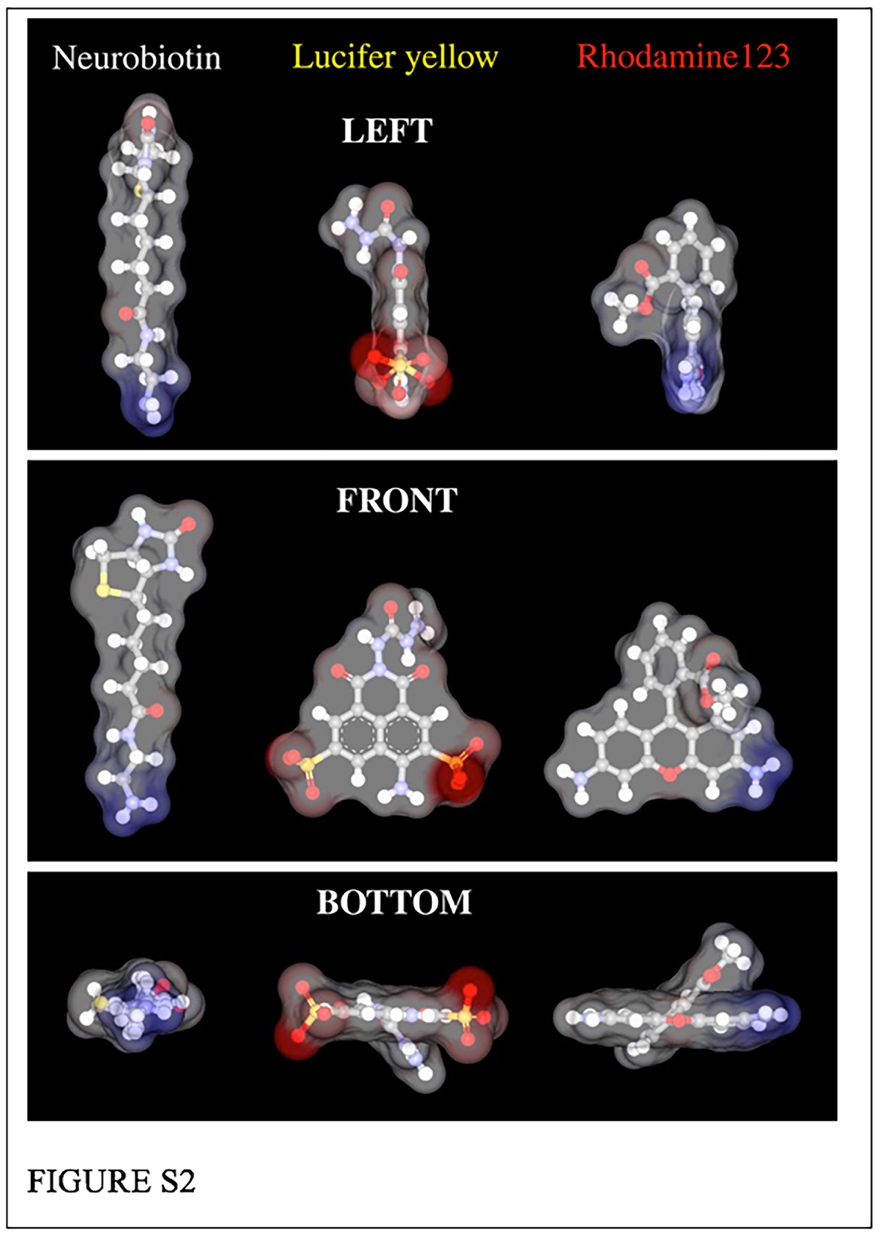
**

**Figure S2**. Molecular representation of Lucifer yellow and Rhodamine 123. Both molecules have a similar molecular weight but their 3D structure show differenced that could account for changes in permeability through Cx45 or Cx43. In a frontal view (left) of both molecules, they appear to have similar areas of interaction, but in a lateral view (on the right) Rhodamine 123 appears larger.
